# Concordant and opposite roles of DNA-PK and the "facilitator of chromatin transcription" (FACT) in DNA repair, apoptosis and necrosis after cisplatin

**DOI:** 10.1186/1476-4598-10-74

**Published:** 2011-06-16

**Authors:** Janna Sand-Dejmek, Guillaume Adelmant, Bijan Sobhian, Anne S Calkins, Jarrod Marto, Dirk J Iglehart, Jean-Bernard Lazaro

**Affiliations:** 1 Department of Cancer Biology, Dana-Farber Cancer Institute, 450 Brookline Avenue, Boston, Massachusetts, 02215, USA; 2 Department of Surgery, Brigham and Women's Hospital, 75 Francis Street, Boston, Massachusetts, 02115, USA; 3 Department of Biological Chemistry and Molecular Pharmacology, Harvard Medical School, 240 Longwood Avenue, Boston, Massachusetts, 02115, USA

## Abstract

**Background:**

Platinum-containing chemotherapy produces specific DNA damage and is used to treat several human solid tumors. Tumors initially sensitive to platinum-based drugs frequently become resistant. Inhibition of DNA repair is a potential strategy to enhance cisplatin effectiveness. After cisplatin treatment, a balance between repair and apoptosis determines whether cancer cells proliferate or die. DNA-dependent protein kinase (DNA-PK) binds to DNA double strand breaks (DSBs) through its Ku subunits and initiates non-homologous end joining. Inhibition of DNA-PK sensitizes cancer cells to cisplatin killing. The goal of this study is to elucidate the mechanism underlying the effects of DNA-PK on cisplatin sensitivity.

**Results:**

Silencing the expression of the catalytic subunit of DNA-PK (DNA-PKcs) increased sensitivity to cisplatin and decreased the appearance of γH2AX after cisplatin treatment. We purified DNA-PK by its Ku86 subunit and identified interactors by tandem mass spectrometry before and after cisplatin treatment. The structure specific recognition protein 1 (SSRP1), Spt16 and γH2AX appeared in the Ku86 complex 5 hours after cisplatin treatment. SSRP1 and Spt16 form the facilitator of chromatin transcription (FACT). The cisplatin-induced association of FACT with Ku86 and γH2AX was abrogated by DNase treatment. In living cells, SSRP1 and Ku86 were recruited at sites of DSBs induced by laser beams. Silencing SSRP1 expression increased sensitivity to cisplatin and decreased γH2AX appearance. However, while silencing SSRP1 in cisplatin-treated cells increased both apoptosis and necrosis, DNA-PKcs silencing, in contrast, favored necrosis over apoptosis.

**Conclusions:**

DNA-PK and FACT both play roles in DNA repair. Therefore both are putative targets for therapeutic inhibition. Since DNA-PK regulates apoptosis, silencing DNA-PKcs redirects cells treated with cisplatin toward necrosis. Silencing FACT however, allows both apoptosis and necrosis. Targeting DNA repair in cancer patients may have different therapeutic effects depending upon the roles played by factors targeted.

## Background

Platinum-containing drugs are used against many solid tumors, are decisive in the treatment of testicular cancer and the main therapy against ovarian cancer, the leading cause of gynecological cancer mortality [1]. Surgery followed by adjuvant platinum-based chemotherapy (e.g., cisplatin or carboplatin) is an effective strategy, but tumors tend to re-occur and cancer returning after initial platinum-based chemotherapy is inevitably resistant to these drugs [2-4]. Platinum salts (hereafter referred to as cisplatin) produce predominantly DNA intra-strand cross-links between adjacent purines (1,2-adducts) that can cause changes in DNA conformation and affect DNA replication and/or gene transcription, resulting in cell-cycle arrest and apoptotic cell death [5-7]. Un-repaired intra- and inter-strand cross-links will eventually result in double-strand DNA breaks (DSBs) at stalled replication forks. Un-repaired cross-links and DSBs will lead to apoptosis which itself produces lethal levels of DSBs [4]. Avoidance of DNA damage-induced cell death by increased DNA repair is a principal mechanism of drug resistance [8,9]. Hence, targeting DNA repair may increase the efficacy of DNA damaging drugs such as cisplatin [10,11]. We discovered that knock down of either the Structure-Specific Recognition Protein 1 (SSRP1) or the catalytic subunit of the DNA-dependent protein kinase (DNA-PKcs) sensitizes transformed cells to cisplatin [12]. The mechanism of increased sensitivity after depletion of SSRP1 or DNA-PKcs may involve effects on cell proliferation, DNA repair or apoptosis.

DNA-dependent protein kinase (DNA-PK) is a serine/threonine kinase required for non-homologous DNA end joining (NHEJ). During NHEJ, the Ku heterodimer, composed of Ku70 and Ku86 proteins, recognizes and binds DNA ends at DSBs. DNA-PKcs is recruited to the DNA-bound Ku heterodimer to form the DNA-PK holoenzyme. The DNA Ligase IV complex, consisting of the catalytic subunit DNA Ligase IV and its cofactor XRCC4, performs the ligation step of repair [13]. DNA-PK is also involved in telomere maintenance, apoptosis and gene transcription, although its precise roles in these processes are not fully characterized [14-16].

The Facilitator of Chromatin Transcription (FACT) is a heterodimer composed of Suppressor of Ty (Spt16) and SSRP1 [17]. SSRP1 is an 87 kDa, high mobility group (HMG) domain-containing protein that binds cisplatin-modified DNA [5,18]. We found that cisplatin induced the exit of DNA-PK and FACT from the nucleolus. DNA-PK activity was necessary for cisplatin-induced loss of nucleolar SSRP1 [12]. Thus, at some level, the functions of FACT and DNA-PK are linked.

In this study we purified the Ku86 complex and showed recruitment of FACT into the complex after cisplatin treatment. FACT and DNA-PK control the appearance of γH2AX on damaged chromatin, co-localize in vivo at the site of DNA damage and contribute to the intrinsic resistance of cancer cells to cisplatin. However, only DNA-PK stimulates the apoptotic response to DNA damage.

## Results

### Dual role of DNA-PK in the response to cisplatin

**Figure 1:**
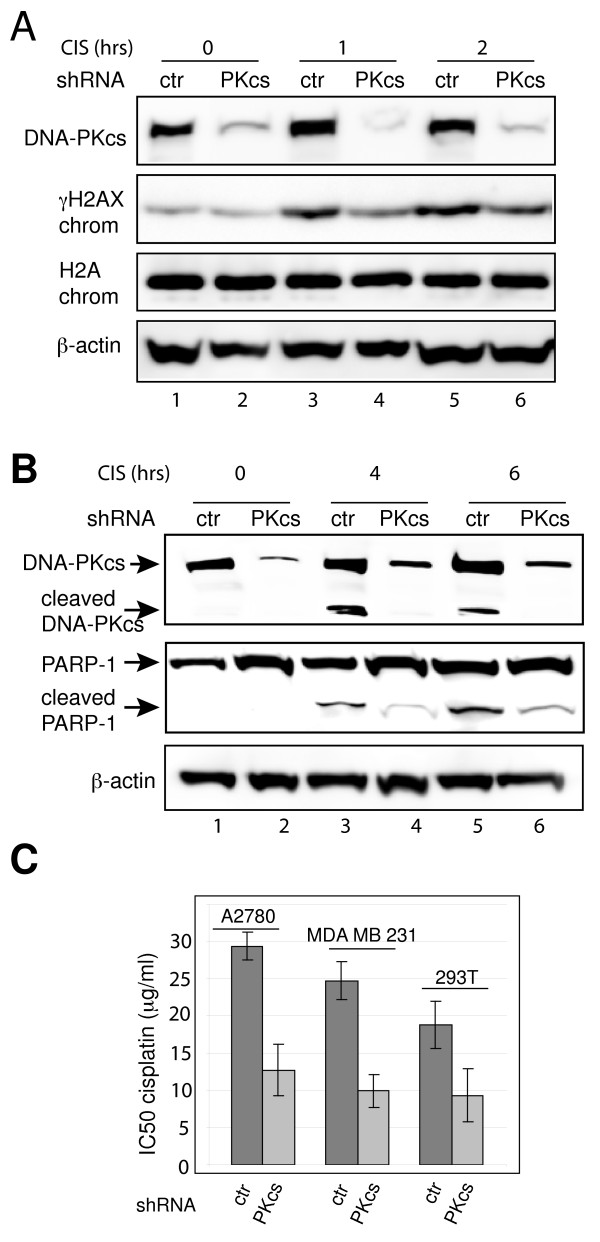
**Dual role of DNA-PK in response to cisplatin**. A. Cisplatin-induced phosphorylation of H2AX is regulated by DNA-PK. DNA-PKcs (PKcs) or control (ctr) shRNA-expressing A2780 cells were treated with 100 μg/ml cisplatin (CIS) for 0, 1 or 2 hours (hrs). DNA-PKcs and β-actin were analyzed in whole cell lysates by immunoblotting. γH2AX and H2A were analyzed in chromatin fractions (chrom). B. Levels of cisplatin-induced PARP-1 cleavage after silencing DNA-PKcs expression in A2780 cells. DNA-PKcs, PARP-1, and β-actin were analyzed in whole cell lysates of cells treated with 100 μg/ml cisplatin for 0, 4 or 6 hrs by immunoblotting. C. DNA-PK inhibition sensitizes cancer cells to cisplatin. Control and DNA-PKcs shRNA-expressing A2780, MDA-MB-231 and HEK293T cells were treated with increasing concentrations of cisplatin. IC_50 _values at 24 hrs are shown. Results represent the mean ± s.e.m. of five independent experiments. For comparisons of control and DNA-PKcs depletion within cell lines, p < 0.01.

Both knock down of DNA-PKcs by shRNA and the DNA-PK inhibitor vanillin sensitize breast cancer cells to cisplatin [12,19]. These results suggest a role for DNA-PK in the repair of cisplatin-induced DNA damage. Phosphorylation of H2AX at serine 139 (γH2AX) is one of the earliest cellular responses to DNA damage and is necessary for triggering DNA repair [20,21]. DNA-PK is capable of phosphorylating nucleosomal H2AX [22]. To monitor the effect of silencing DNA-PKcs expression on the induction of DNA repair, we monitored the amounts of γH2AX during the first 2 hours following cisplatin treatment of human ovarian cancer A2780 cells (Figure 1A). Immunoblotting chromatin fractions from cells expressing control shRNA revealed γH2AX one hour after cisplatin treatment. However, levels of γH2AX were reduced by 37.0% and 34.6% respectively 1 and 2 hours after treatment in cells silenced for DNA-PKcs expression (Figure 1A). Persistent phosphorylation of H2AX after silencing DNA-PKcs is likely due to other kinases involved in DNA damage repair [23,24].

DNA-PK also participates in the apoptotic response to DNA damage [14]. We monitored apoptosis in A2780 cells treated with cisplatin by analysis of cleaved poly(ADP-Ribose) polymerase 1 (PARP-1) protein levels. Immunoblotting showed that depletion of DNA-PKcs reduced PARP-1 cleavage in cells treated with 100 μg/ml cisplatin as soon as 4 hours after cisplatin addition (Figure 1B, compare lanes 3 and 4). Notably, DNA-PKcs itself was a target for apoptosis-induced cleavage (Figure 1B, lanes 4 and 6). Similar results were obtained with the MDA-MB-231 (human breast cancer) and HEK293T (human embryonic kidney) cell lines expressing shRNA against DNA-PKcs (data not shown). These results suggest a role for DNA-PK in controlling the induction of apoptosis by cisplatin.

Since DNA-PK is involved in two opposing functions, DNA repair and apoptosis, we examined the effects of DNA-PKcs depletion on cisplatin-induced cytotoxicity. We treated A2780, MDA-MB-231 and HEK293T cells expressing an shRNA directed against DNA-PKcs or a control shRNA with cisplatin (Figure 1C). DNA-PKcs expression was reduced by more than 75% in the A2780, MDA-MB-231 and HEK293T cell lines (data not shown). Cells expressing shRNA directed against DNA-PKcs were more sensitive to cisplatin treatment than cells expressing control shRNA with an IC_50 _significantly below that of control cells (Figure 1C and Additional file 1). The concentration range of cisplatin used in the above experiments spanned 1 to 100 μg/ml. Bromodeoxyuridine (BrdU) incorporation assays at each concentration showed 100% inhibition of the S-phase counts in all cell lines, ruling out the possibility that differences in cell proliferation due to depletion of DNA-PKcs influenced our results (data not shown). Monitoring the inception of DNA repair and apoptosis shortly after cisplatin treatment establishes that DNA-PKcs regulates cell survival but plays roles apparently opposite in promoting both DNA repair and apoptosis.

### DNA-dependent interaction of FACT with the Ku complex

**Figure 2:**
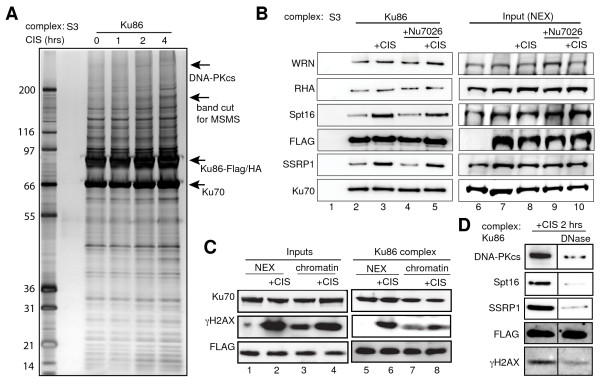
**Cisplatin-induced recruitment of FACT to the Ku complex**. A. Purification of the Ku86 complex. Tandem affinity purification for FLAG and HA was performed on nuclear extracts from S3 or S3-Ku86-Flag/HA cells 0, 1, 2 and 4 hours (hrs) after cisplatin (CIS) treatment. The purified complexes were resolved by 4-12% NuPAGE and visualized by silver staining. Arrows show Ku70, Ku86-Flag/HA, the band identified as spt16 through MSMS analysis and DNA-PKcs (based on molecular weight). The molecular weight markers are indicated on the left (in kDa). B. Association of FACT with the Ku complex. Complexes purified as in (A) before or 4 hrs after cisplatin treatment (+CIS) were immunoblotted for the indicated proteins. Cells were pre-treated with the DNA-PK inhibitor Nu7026 as indicated. C. Cisplatin-induced association of γH2AX with the Ku complex. Inputs are nuclear extracts (NEX) and chromatin obtained before or 4 hrs after cisplatin treatment (+CIS). Inputs and Ku86 complexes purified from the inputs were immunoblotted for the indicated proteins. D. Treatment of the Ku complex with DNase reveals DNA-dependent interactions. Ku86 complexes were purified as in (A) 2 hrs after cisplatin treatment. The complexes immobilized on anti-HA beads were left untreated or treated with DNase and then eluted with the HA peptide. The resulting complexes were analyzed by immunoblotting for the indicated proteins.

To understand the dual role of DNA-PK in controlling both repair and apoptosis we purified the DNA-PK complex using tagged Ku86 as bait. We hypothesized that changes in the composition of the Ku protein complex after cisplatin treatment will provide insights into the cellular response to cisplatin-induced DNA damage. Nuclear extracts of HeLa S3 cells stably expressing Ku86-Flag/HA were submitted to sequential Flag and HA immunoprecipitations [25]. Cleaved caspase-3 and cleaved DNA-PKcs were detected between 2 and 4 hours after the beginning of cisplatin treatment in S3 cells (Additional file 2). Therefore complexes were purified from untreated cells and 1, 2 and 4 hours after cisplatin treatment. Silver stain of the complexes showed Ku86 co-precipitates with stoïchiometric amounts of its heterodimeric partner Ku70 and a number of other proteins. Although noticeable changes in the subunit composition of the complex appeared 2 hours after cisplatin treatment, they were more pronounced after 4 hours (Figure 2A). In three different experiments, a polypeptide appeared approximately at 140 kDa in the Ku86 complex 4 hours after cisplatin treatment (see arrow, Figure 2A). The corresponding band was excised from the gel shown in Figure 2A and submitted to tandem mass spectrometry (MSMS) analysis. This band contained, among others, a major polypeptide identified as the FACT subunit Spt16 (Table 1).

**Table 1:** Proteins identified by LC-MS/MS of cut bands in the Ku86 complex

**Protein name**^**1**^	**Synonym**^**1**^	**Accession Number**^**1**^	**Theoretical MW (kDa)**^**1**^	**No. of Peptides**^**2**^	**Function**^**3**^
ATP-dependent DNA helicase 2 subunit 1	Ku70	P12956	69.71	21	DNA repair

ATP-dependent DNA helicase 2 subunit 2	Ku86	P13010	82.57	20	DNA repair

FACT complex subunit SPT16^4^	SPT16	Q9Y5B9	119.91	11	Nucleosome/transcription

Werner syndrome ATP-dependent helicase	WRN	Q14191	162.49	7	Helicase

Lamina-associated polypeptide 2 isoform alpha	LAP2A	P42166	75.36	7	DNA binding

DNA topoisomerase 1	TOP1	P11387	90.73	1	Chromatin binding

Poly (ADP-ribose) polymerase 1	PARP-1	P09874	112.95	1	DNA binding

ATP-dependent RNA helicase	DDX17	Q92841	72.37	1	Helicase

^1 ^as in the SWISS-PROT database, ^2 ^number of peptide obtain by LC-MSMS analysis of the unique silver stained band shown in figure 1, ^3 ^as in the Gene Ontology classification.

Spt16 associates with SSRP1 to form the FACT complex. SSRP1 migrates at 87 kDa and could be masked by the large Ku86 band in Figure 2A. Spt16 and SSRP1 protein levels were analyzed in Ku86 complexes purified before and after cisplatin. Both proteins were detected in the complex from untreated cells and a significant cisplatin-induced increase was observed (Figure 2B lanes 2 and 3). Levels of other Ku-associated proteins, including WRN and RHA, remained unaffected by cisplatin, suggesting specificity in the recruitment of FACT to the Ku complex (Figure 2B, lanes 2 and 3).

We previously observed that the specific DNA-PK inhibitor Nu7026 prevented cisplatin-induced loss of the nucleolar fraction of SSRP1 [12]. Therefore, we wondered whether the cisplatin induced recruitment of FACT to the Ku complex could be prevented by pretreatment of the cells with Nu7026. Our results show no change in the cisplatin-induced recruitment of FACT after DNA-PK inhibition (Figure 2B, lanes 4 and 5). Therefore, recruitment of FACT after cisplatin treatment does not require the kinase activity of DNA-PK. The overall amounts of these proteins in the nuclear extracts used for immunoprecipitation were not affected by cisplatin, indicating changes in the concentration of FACT in the Ku complex were not due to changes in the overall nuclear amounts of FACT (Figure 2B; lanes 7, 8, 9 and 10).

Heo and colleagues reported the association of FACT and DNA-PK on nucleosomes containing H2AX [26]. Since FACT associated with the Ku86 complex purified from nuclear extracts after cisplatin-induced DNA damage (Figure 2A and 2B), we asked whether γH2AX is present in such a complex. Four hours after cisplatin treatment, γH2AX levels were up-regulated in nuclear extracts and chromatin fractions prior to purification of the Ku86 complex (Figure 2C, input panel). Similarly, γH2AX was detected in the Ku86 complex purified from nuclear extracts of S3-Ku86-Flag/HA cells treated with cisplatin, but not in complexes from untreated cells (Figure 2C; lanes 5 and 6). Analysis of chromatin fractions shows enrichment of γH2AX in the Ku complex after cisplatin (Figure 2C; lanes 7 and 8). Hence, increased γH2AX levels in the Ku complex reflect the overall appearance of γH2AX after cisplatin treatment. H2AX is phosphorylated on chromatin (i.e., when part of nucleosomes). This suggests nucleosomes issued from chromatin fragmentation in the nuclear extracts from cisplatin treated cells are the source of γH2AX in Ku complexes found in the nuclear extracts.

Both DNA-PK and FACT have DNA binding properties [18,27]. Therefore, DNA-PK and FACT could interact in a ternary complex with DNA. To investigate whether the interaction between DNA-PK and FACT was DNA-dependent, we analyzed the effects of DNA nuclease (DNase) treatment on the composition of the Ku86 complex purified 2 hours after cisplatin. Although γH2AX, SSRP1, Spt16 and DNA-PKcs were readily detected in the un-treated Ku86 complex, the association of these proteins with Ku86 was abolished by DNase treatment (Figure 2D). These results suggest that cisplatin provokes the DNA-dependent association of DNA-PK with FACT and that DNA-PK and FACT do not interact directly.

### FACT and DNA-PK associate at sites of DNA damage in situ

**Figure 3:**
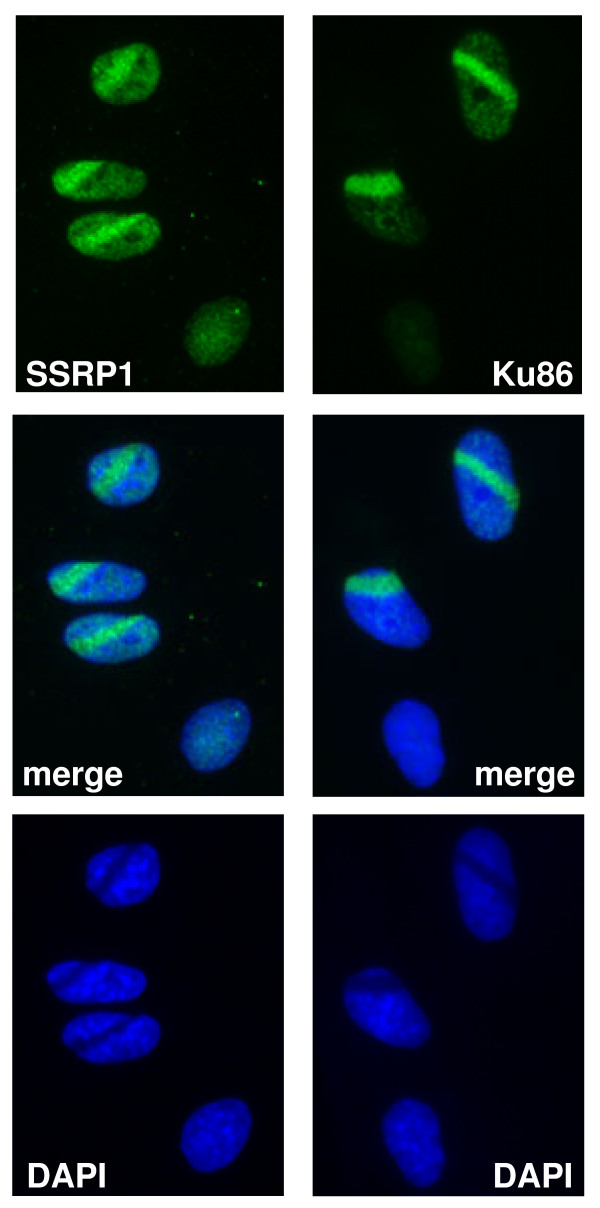
**Ku86 and SSRP1 co-localize with DSBs**. Cells were fixed with paraformaldehyde and methanol 50 to 60 minutes after DSBs induction using a λ = 337 nm "MicroBeam" laser. The cells were immunostained for SSRP1 or Ku86 as indicated. Nuclei were visualized with DAPI.

We wondered if Ku86 and FACT localize together onto damaged DNA. Bathing cells in a solution of cisplatin damages cellular DNA in a spatially unrestricted way and does not allow production of damage at discrete points in nuclei. In order to induce damage in a spatially restricted way, we produced double strand breaks (DSBs) with a UV laser designed for micro-dissection. A2780 cells treated overnight with BrdU were laser-damaged. Ku86 and SSRP1 localization were analyzed by immunofluorescence. Sites of damage were easily seen as dark stripes cutting through DAPI stained nuclei in cells fixed 60 minutes after damage (Figure 3, lower panels). Both SSRP1 and Ku86 were recruited to stripes of DSBs (Figure 3). Similar results were obtained showing recruitment of DNA-PKcs to DSBs (data not shown).

Ku and DNA-PKcs are effectors of NHEJ. Homologous recombination (HR)-associated proteins and γH2AX are also recruited to stripes of DSBs. Since γH2AX and HR-associated proteins form DNA damage-induced foci [28], we wondered if SSRP1 and Ku86 aggregate with such foci. We produced DNA damage-induced foci by γ-irradiation or cisplatin treatment and did not observe co-localization of SSRP1 and Ku86 with γH2AX (Additional file 3 and 4) when γH2AX formed foci with BRCA1 (Additional file 5). These results demonstrate that DNA-PK and FACT are present together on damaged DNA in living cells and suggest that FACT may not participate in HR.

### FACT does not share the dual role of DNA-PK in response to cisplatin

**Figure 4:**
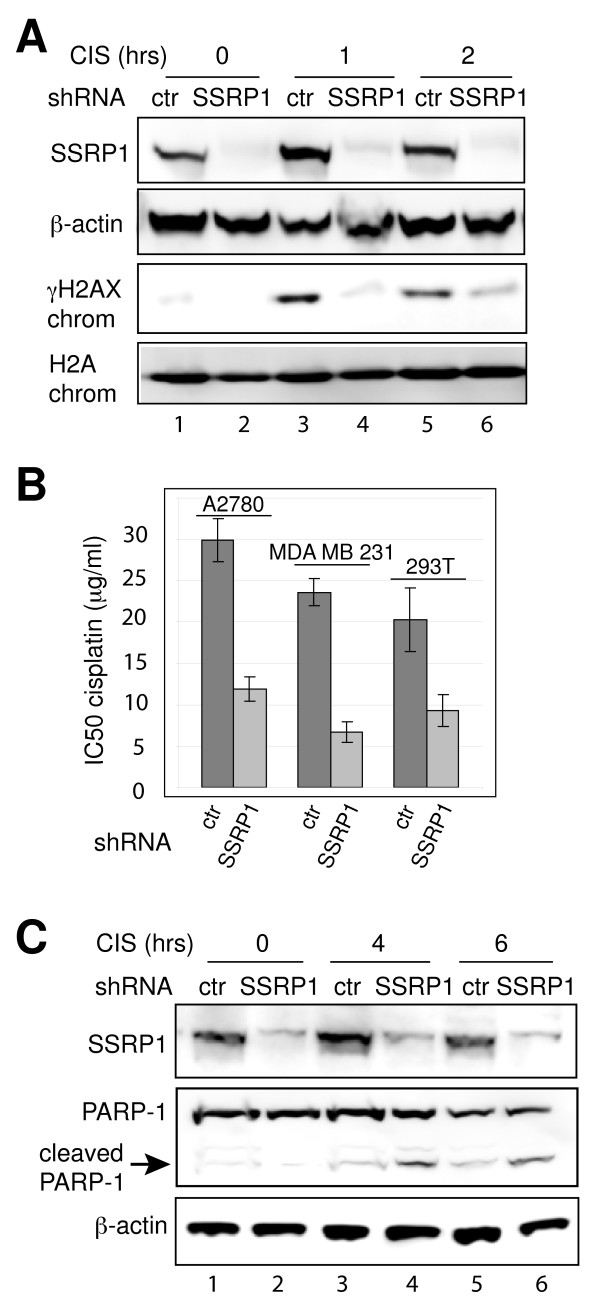
**Role of FACT in response to cisplatin**. A. Cisplatin-induced appearance γH2AX is SSRP1-dependent. SSRP1 or control (ctr) shRNA-expressing A2780 cells were treated with 100 μg/ml cisplatin for 0, 1 or 2 hrs. Using immunoblotting SSRP1 and β-actin were detected in whole cell lysates and γH2AX and H2A in chromatin fractions. B. FACT inhibition sensitizes cancer cells to cisplatin. SSRP1 and control shRNA-expressing A2780, MDA-MB-231 and HEK293T cells were treated with increasing concentrations of cisplatin. IC_50 _values at 24 hrs are shown. Results represent the mean ± s.e.m. of five independent experiments. For comparisons of control and SSRP1 depletion within cell lines, p < 0.01. C. Levels of cisplatin-induced PARP-1 cleavage after silencing SSRP1 expression in A2780 cells. SSRP1, PARP-1, and β-actin were analyzed in whole cell lysates of cells treated with 100 μg/ml cisplatin for 0, 4 or 6 hrs by immunoblotting.

DNA-PK has a dual role, participating in both repair and apoptosis after cisplatin treatment (Figure 1). Therefore, we investigated the role of FACT in repair and apoptosis. We established A2780 cell lines expressing shRNA against SSRP1, or control shRNA (Figure 4A, lanes 1 and 2) and analyzed γH2AX levels after cisplatin treatment. After treatment with 100 μg/ml cisplatin for 1 or 2 hours γH2AX was readily detected in chromatin of control cells (Figure 4A, lanes 3 and 5). In contrast SSRP1 depletion reduced the cisplatin-induced appearance of γH2AX by 86.8% and 60.0% respectively 1 and 2 hours after treatment (Figure 4A, lanes 4 and 6). These results were consistent with the reported role of FACT in H2AX exchange at the nucleosome [26].

Knock down of SSRP1 expression in A2780, MDA-MB-231 and HEK293T cells increased sensitivity to cisplatin (decreased the IC_50 _value) when compared to cells expressing control shRNA (Figure 4B). These effects could be due to SSRP1 regulation of DNA-PK activity. However, silencing of SSRP1 expression did not prevent the cisplatin-induced autophosphorylation of DNA-PKcs at serine 2056 (Additional file 6A). These results rule out the regulation of DNA-PK kinase activity by FACT. Furthermore SSRP1 knock down did not alter protein expression levels of Ku86, DNA-PKcs or Spt16 (Additional file 6B). It is likely SSRP1 plays a singular and direct role in DNA repair after cisplatin-induced damage.

We then performed a comparative analysis of apoptosis in control and SSRP1-depleted cells after cisplatin treatment. Higher levels of cleaved PARP-1 were detected in SSRP1-depleted A2780 cells treated with cisplatin (Figure 4C, lanes 4 and 6) when compared to control cells (Figure 4C, lanes 3 and 5). Similar results were obtained when SSRP1 knock down effects were analyzed in the MDA-MB-231 breast cancer cells (Additional file 6C). These results contrast with DNA-PKcs knock down shown in Figure 1B and suggest SSRP1 depletion increased the apoptotic response to cisplatin.

### Regulation of apoptosis and necrosis by DNA-PK and FACT after cisplatin

Despite the implication of DNA-PK in the execution of apoptosis (Figure 1B), cisplatin cytotoxicity is increased in cells with depleted DNA-PKcs (Figure 1C and Additional file 1). Cisplatin can induce apoptosis as well as necrotic cell death [29].

**Figure 5:**
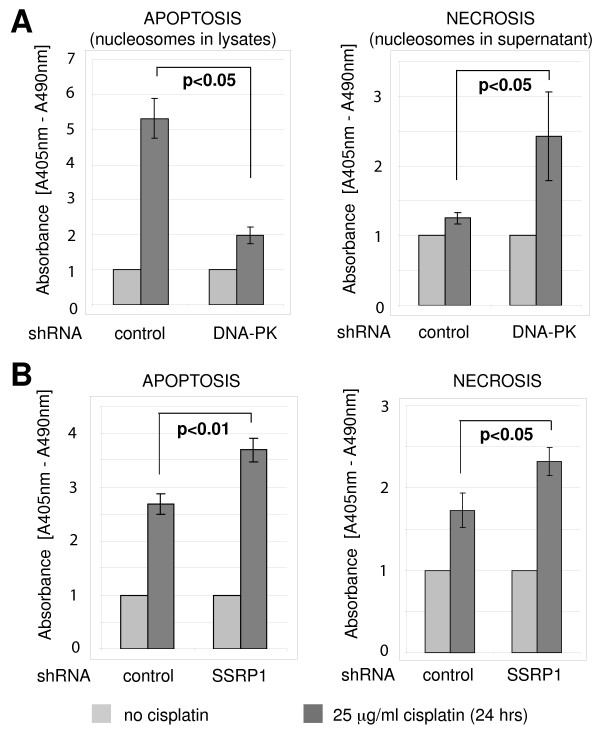
**Differential regulation of cisplatin-induced apoptosis and necrosis by DNA-PK and FACT**. A. Regulation of cisplatin-induced apoptosis and necrosis by DNA-PKcs. DNA-PKcs and control shRNA-expressing A2780 cells were treated with 25 μg/ml cisplatin for 24 hrs and relative amounts of nucleosomes were quantified in lysates (apoptosis) and culture supernatants (necrosis). Comparison between cisplatin treated control and DNA-PKcs depleted cells is shown, p < 0.05. B. Regulation of cisplatin-induced apoptosis and necrosis by SSRP1. SSRP1 and control shRNA-expressing A2780 cells were treated and analysed as in (A). Comparison between cisplatin treated control and SSRP1 depleted cells, p < 0.01 (apoptosis) and p < 0.05 (necrosis) are shown.

Chromatin fragmentation is induced by both apoptosis and necrosis. However, necrosis results in cell membrane fragmentation and subsequent release of fragmented chromatin (free nucleosomes) in the culture supernatant. Because apoptosis does not induce fragmentation of the cell membrane, cells must first be lysed to extract free nucleosomes. We quantified levels of free nucleosomes in supernatants and lysates of A2780 cells expressing DNA-PKcs, SSRP1 or control shRNA and treated with different concentrations of cisplatin, ranging from 1.5 to 25 μg/ml for 4 hours (Figure 5, Additional file 7). In control cells, appearance of nucleosomes in lysates after cisplatin treatment indicated robust apoptosis with little evidence for necrosis (Figure 5, and Additional file 7). Depletion of DNA-PKcs reduced apoptosis after cisplatin (p < 0.05; Figure 5A and Additional file 7A). After DNA-PKcs depletion, necrosis became the dominant response to cisplatin (p < 0.05; Figure 5B and Additional file 7A). In contrast to DNA-PKcs depletion, cisplatin-induced apoptosis was higher in cells lacking SSRP1 than in control cells (P < 0.01; Figure 5C and Additional file 7B). Although to a lesser extent than in cells lacking DNA-PKcs, levels of cisplatin-induced necrosis increased in SSRP1-depleted cells when compared to control cells (P < 0.05; Figure 5D and Additional file 7B).

Consistent with the inhibition of DNA repair, depletion of SSRP1 increases both apoptosis and necrosis in response to cisplatin. Because DNA-PK participates in the initiation of apoptosis, depletion of DNA-PKcs redirects cisplatin-treated cells toward necrosis. These results explain why similar levels of cisplatin-induced cytotoxicity are observed after silencing DNA-PKcs or SSRP1. In conclusion FACT participates with DNA-PK in the DNA repair response, but FACT does not share the apoptotic functions of DNA-PK.

## Discussion

Platinum-containing drugs react with DNA to form adducts that must be excised, and the subsequent breach in DNA repaired, in order to avoid cell death through apoptosis. There is both direct and circumstantial evidence that proficiency in DNA repair explains in part the sensitivity of cancers to platinum-based chemotherapy [9,11,30]. Testicular cancers, which are very sensitive to cisplatin, are deficient in repair, whereas other solid tumors are more proficient in repair and less sensitive to platinum [4]. Breast and ovarian cancer cells lacking function of either the BRCA1 or BRCA2 susceptibility gene products are deficient in homologous recombination and more sensitive to platinum-containing drugs [31-33]. The excision repair cross complementation group 1 (ERCC1) is deficient in some lung cancers, and patients with deficient tumors are more sensitive to cisplatin-based therapy than tumors with sufficient ERCC1 [34]. Perhaps the strongest evidence for the role of DNA repair in resistance to cisplatin is the somatic reversion of BRCA1 and BRCA2 mutations to DNA repair proficient proteins in chemotherapy-resistant cancers, initially treatment-sensitive [33,35,36]. Success of cisplatin therapy depends on ability of cancer cells to repair cisplatin damage. Treatment of solid tumors partially crippled by DNA repair deficiencies opens a therapeutic window of opportunity. This opportunity, called synthetic lethality is a promising strategy for treatment of cancers with DNA repair deficiencies [8,37]. Indeed, clinical trials testing the principle of synthetic lethality caused by BRCA1 and BRCA2 deficiencies, using PARP-1 inhibitors are underway [38-40]. However, for patients without inherited defects in DNA repair pathways, the combination of disabling components of repair with genotoxic chemotherapy is logical [41].

PARP-1, FACT and DNA-PK co-purify in the H2AX complex suggesting a coordinated role during DNA repair [24]. We examined the effect of the inhibition of DNA-PK and FACT on cytotoxicity due to cisplatin. Cisplatin cytotoxicity is enhanced by vanillin, a natural inhibitor of DNA-PK activity [19] and by shRNA reduction of DNA-PKcs (Figure 1C, Additional file 1 and [12]). Disabling FACT by depletion of SSRP1 also sensitizes cells to cisplatin (Figure 4B, Additional file 1 and [12]). These results corroborate the hypothesis that disabling DNA repair can be combined with DNA damage to induce synthetic lethality.

We looked more closely at DNA-PK and FACT after cisplatin treatment. SSRP1 was identified screening a human cDNA expression library for proteins that specifically bound cisplatin-modified DNA [5]. Additionally, we show FACT is necessary for the full expression of γH2AX, co-localizes with DNA-PK at the site of DNA damage and is co-purified with Ku86 in a DNA-dependent manner. DNA also stabilizes the association of DNA-PKcs with the Ku heterodimer [42]. The Ku complexes containing DNA-PKcs and FACT were purified from nuclear extracts after cisplatin treatment. Nucleosomes are found in nuclear extracts when chromatin fragmentation occurs (e.g. during apoptosis). Hence, FACT probably is associated with Ku on nucleosomes freed by DNA fragmentation during cell apoptosis. We therefore investigated the association of DNA-PK and FACT with damaged DNA, in living cells prior to apoptotic fragmentation.

We spatially restricted DNA DSBs using low energy laser light after sensitization with BrdU and found Ku86 and SSRP1 localized to DSBs (Figure 3). Ku86 and SSRP1 presence at DSBs seems unrelated to HR since they were not recruited to γH2AX/BRCA1 foci after γ-irradiation or cisplatin treatment (Additional files 3, 4 and 5). We previously showed that loss of DNA-PKcs prevents the cisplatin-induced exit of FACT from the nucleolus [12]. Therefore, a model of events after cisplatin damage includes mobilization of DNA-PK and FACT from the nucleolus, association with damaged chromatin, and initiation of DNA repair. Disabling these events by inhibiting or depleting subunits of FACT and DNA-PK complexes accentuates the cytotoxicity of cisplatin, probably by hindering DNA repair.

Furthermore, our work suggests the possibility of calibrating the inhibition of DNA repair by carefully choosing the molecular target. Cells with stable DNA-PKcs knock down are more sensitive to cisplatin despite a two-fold reduction in the level of apoptosis at each dose of cisplatin (Figure 1 and Additional file 1 and 7). Both apoptosis and necrosis occur in cisplatin-treated cells [6,29]. Recent findings indicate that necrosis may be a default cell death pathway that is unmasked when essential factors of apoptosis are inhibited [43]. We found an increase in cisplatin-induced necrosis after knock down of DNA-PK as well as FACT. However, only FACT knock down was associated with both apoptosis and necrosis (Figure 5). Thus increased sensitivity to DNA damage after DNA-PK inhibition can be explained by an increase in necrosis.

DNA-PK was reported necessary for the activation of apoptosis by etoposide [44] and in mouse thymocytes and fibroblasts, p53-dependent apoptosis induced by ionizing irradiation is suppressed in the absence of DNA-PK [45,46]. Hence, DNA-PK is at a central fork in cell fate after DNA damage, including after cisplatin treatment of cancer cells (Figure 5). Practically, DNA-PK is a "drug-able" kinase and DNA-PK inhibitors might conveniently be combined with cisplatin chemotherapy.

## Conclusions

Because of its many roles, the consequences of inhibiting DNA-PK are difficult to predict when compared to inhibition of proteins involved in simpler linear pathways. FACT is necessary for phosphorylation of H2AX (Figure 4A) and likely the subsequent repair of damaged DNA [26]. Silencing SSPR1 has no effect on activation of DNA-PK (Additional file 6A) and stimulates apoptosis in cisplatin-treated cells (Figure 4C and 5B). Hence, inhibiting FACT may have different consequences than inhibiting DNA-PK. Future pre-clinical studies in animals will determine which target in DNA repair pathways opens the most promising therapeutic window in combination with cisplatin. In conclusion, although inhibition of DNA repair during cisplatin treatment is a rational combination, depending upon the DNA repair target chosen, the effects may be quite different.

## Methods

### Cell lines

Human MDA-MB-231 breast and A2780 ovarian adenocarcinoma cells (ATCC, Manassas, VA) were cultured in RPMI medium containing 10% (v/v) fetal bovine serum, penicillin and streptomycin (Invitrogen, Carlsbad, CA). HeLa S3 (S3) cells and derivatives, HEK293T cells and Linx cells (Open Biosystems, Thermo Fisher Scientific, Huntsville, AL) where cultured in DMEM containing 10% (v/v) calf serum (Invitrogen) and antibiotics.

### Chemicals

Cisplatin (*cis*-diamminedichloro-platinum) powder (Sigma-Aldrich, St Louis, MO) was prepared freshly in cell culture medium. Nu7026 (Sigma) was dissolved in DMSO and stored at -20 C.

### Antibodies

Mouse monoclonal antibodies used were anti-DNA-PKcs 18-2, anti-Ku86 111 and anti-Ku70 N3H10 (Neomarkers/Labvision, Thermo Fisher Scientific), anti-FLAG M2 (Sigma-Aldrich), anti-SSRP1 and anti-SPT16 (Biolegend, San Diego, CA), anti-phospho(ser139)-H2AX clone JBW301 (Abcam, Cambridge, MA), anti-Poly (ADP ribose) Polymerase-1 (PARP-1, BD Biosciences, San Jose, CA). Rabbit antibodies were anti-RHA, anti-WRN (Santa Cruz Biotechnology, Santa Cruz, CA), anti-H2A (Upstate, Millipore, Billerica, MA), anti-phospho(ser139)-H2AX (Abcam), anti-cleaved caspase-3 (Cell Signaling Technology, Danvers, MA) and anti-DNA-PKcs phospho-serine 2056 (a kind gift from Dr. B. Chen) [47].

### Immunofluorescence analysis

Cells grown on glass coverslips were fixed with 3.7% paraformaldehyde (PFA) and permeabilized with methanol for 10 minutes. The cells were incubated with primary antibodies at a 1/300 dilution for 1 hr at 37C, rinsed with PBS and incubated for 30 min at 37C with secondary antibodies conjugated to Alexa Fluor-488 or Alexa Fluor-594 fluorochromes (Molecular Probes, Invitrogen), at a 1/300 dilution. DNA was visualized by DAPI (Sigma-Aldrich). Fluorochromes were visualized with an Axioskop II microscope and imaged with AxioVision 4.5 software (Zeiss, Jena, Germany).

### Assays of DNA damage

Laser-induced DNA DSBs were generated using a P.A.L.M. MicroBeam laser microdissection system (Zeiss) at λ = 337 nm as previously described [48,49]. Cells were grown on coverslips for 24 hours in media containing 10 uM BrdU (Sigma-Aldrich) prior to laser treatment. After laser stripe generation, cells were incubated at 37°C and fixed 60 min later for immunofluorescence. DNA damage-induced foci were generated either by γ-irradiation or cisplatin and visualized by γH2AX immunofluorescence [50].

### Plasmids and transfection

For DNA-PKcs silencing, retroviruses were produced by transfecting retroviral pSM2c expression vector (Open Biosystems) containing a puromycin resistance gene and a control shRNA (5'TCTCGCTTGGGCGAGAGTAAG) or a shRNA to DNA-PKcs (5'GGAGCTTACATGCTAATGTAT) into the Linx packaging cell line. On day three, virus-containing supernatants were added to MDA-MB-231 and A2780 cells and incubated in 5 μg/ml polybrene. For knock down of FACT, small hairpin sequences specific to SSRP1 (5'CACCACAGTACTGCGTCTGTT) were cloned into the pcDNA6.2 vector (Invitrogen) containing a blastocidin resistance gene, according to manufacturer instructions. A control shRNA sequence (5'GTCTCCACGCGCACTACATTT) was used to generate a non-silencing control plasmid. Transfection of HEK293T, MDA-MB-231 and A2780 cells was assisted by FuGENE HD (Roche, Indianapolis, IN). Selection with puromycin or blastocidin started 48 hrs after transfection. After 10 days resistant colonies where expanded and protein knock down determined by immunoblotting and immunofluorescence.

### Tandem affinity purifications

HeLa S3 cells expressing Ku86 fused to Flag and HA tags (S3-Ku86-Flag/HA) were treated as indicated. Sub-cellular fractions were prepared as described [51]. Briefly, cells were incubated in hypotonic buffer (10 mM Tris-HCl [pH 8], 10 mM KCl, 1.5 mM MgCl2) on ice for 10 min and homogenized by tight dounce. Nuclei were collected by centrifugation at 2000 g for 15 min at 4°C and extracted with 40 mM Tris-HCl [pH 8], 200 mM NaCl, 10% glycerol, 2 mM EDTA, 0.5% NP40 and a 1 × protease inhibitor mix for 45 min at 4°C. The insoluble material (chromatin) was pelleted at 15,000 g for 30 min at 4°C, and the supernatant called "nuclear extract" (NEX). NEX or chromatin were further treated through two sequential FLAG and HA immunoprecipitations as previously described [25].

### Chromatin preparation

Chromatin pellets obtained as described above were washed in 20 mM Tris-HCL pH 7.5, 100 mM KCl, 2 mM MgCl_2_, and 1 mM CaCl_2 _and incubated at room temperature in 0.05 U/μl micrococcal nuclease (MNase, Nuclease S7, Roche) for 15 min, pelleted and the supernatant was collected.

### Immunoblotting

Samples were separated on NuPAGE 4-12% gels (Invitrogen), analyzed by immunoblotting with the indicated antibodies and visualized with Supersignal chemi-luminescent reagents (Pierce, Thermo Fisher Scientific) and a luminescent image analyzer LAS-3000mini (Fujifilm, Edison, NJ). When indicated relative amounts of proteins were compared using ImageJ software.

### Cytotoxicity assay

Cytotoxicity was assessed by the 3-(4,5-dimethylthiazol-2-yl)-5-(3-carboxymethoxyphenyl)-2-(4-sulfenyl)-2H-tetrazolium, inner salt (MTS) assay using the CellTiter 96 Aqueous One Solution Proliferation Assay (Promega, Madison, WI), according to the manufacturer's protocol. Cells were seeded in 96-well plates at a density of 5000 cells/well. After overnight incubation, cisplatin was added at the concentrations indicated. The absorbance of each well was measured at 490 nm. Values for control cells were considered as 100% viability. The dose-response curves were plotted as a percentage absorbance of control cells. The half maximal inhibition (IC50) value was calculated from the percent inhibition curve generated using Excel XLfit software (Microsoft, Redmond, WA).

The Cell Death Detection ELISA Assay (Roche) was used to analyze apoptosis and necrosis in response to cisplatin. The assay is a sandwich-enzyme-immunoassay using antibodies directed against DNA and histones and allowing quantification of nucleosomes. Nucleosomes were quantified either in the cell culture supernatant (necrosis) or in cell lysates (apoptosis). The assay was performed as recommended by the supplier.

### Statistical analysis

For the MTS and Cell Death Detection ELISA assays, all values were expressed as mean ± s.e.m. Differences between groups were tested for statistical significance using Student's paired t-test. P < 0.05 represented a significant difference.

## Competing interests

The authors declare that they have no competing interests.

## Authors' contributions

JSD established the molecular knock-down cell lines, performed apoptosis, cell survival and DNA repair studies, purified and analysed the Ku86 complex and co-drafted the manuscript. BS conceived and performed part of the γ-H2AX study. GA and JM designed and performed the DNase experiment. ASC and JBL performed immunofluorescence experiments. ASC performed densitometry analysis of immunoblots. JDI and JBL conceived of the study, and participated in its design and coordination and co-drafted the manuscript. All authors read and approved the final manuscript.

## Supplementary Material

Additional file 1 **Silencing the expression of DNA-PKcs or SSRP1 increases cell sensitivity to cisplatin**. DNA-PKcs, SSRP1 and control shRNA-expressing A2780, MDA-MB-231 and HEK293T cells were treated with increasing concentrations of cisplatin as indicated. Cytotoxicity was assessed by the MTS assay. Click here for file

Additional file 2 **DNA-PK Regulates cisplatin-induced apoptosis**. Extracts from HeLa-S3 cells treated with 100 μg/ml cisplatin for the indicated time were immunoblotted for DNA-PKcs, Ku86 and cleaved caspase-3. Click here for file

Additional file 3 **SSRP1 does not co-localize with DNA damage-induced γH2AX foci**. A2780 cells were immunostained for SSRP1 and γH2AX before or 4 hours after DNA damage. Nuclei were visualized with DAPI. A. Undamaged cells. B. Gamma irradiation (10 Gray). C. Cisplatin treatment (25 μg/ml). Click here for file

Additional file 4 **Ku86 does not co-localize with DNA damage-induced γH2AX foci**. A2780 cells were immunostained for Ku86 and γH2AX before or 4 hours after DNA damage. Nuclei were visualized with DAPI. A. Undamaged cells. B. Gamma irradiation (10 Gray). C. Cisplatin treatment (25 μg/ml). Click here for file

Additional file 5 **BRCA1 co-localizes with DNA damage-induced γH2AX foci**. A2780 cells were immunostained for BRCA1 and γH2AX before or 4 hours after DNA damage. Nuclei were visualized with DAPI. A. Undamaged cells. B. Gamma irradiation (10 Gray). C. Cisplatin treatment (25 μg/ml). Click here for file

Additional file 6 **Effects of SSRP1 depletion on DNA-PK and apoptosis**. A. DNA-PK activation after cisplatin treatment is SSRP1-independent. DNA-PKcs was immunoprecipitated from SSRP1 and control shRNA expressing A2780 cells treated with 100 μg/ml cisplatin for 0, 1 and 2 hours. Immunoprecipitates were analyzed by immunoblotting for DNA-PKcs-Ser2056 and DNA-PKcs. B. SSRP1 knock down does not alter Ku86, Spt16 and DNA-PKcs expression. Whole cell lysates of SSRP1 and control shRNA-expressing A2780 cells were analyzed by immunoblotting for β-actin, Ku86, SSRP1, Spt16 and DNA-PKcs. C. Role of FACT in cisplatin-induced apoptosis in MDA-MB-231 breast cancer cells. Whole cell lysates of SSRP1 or control shRNA-expressing cells treated with 100 μg/ml cisplatin for 0, 4 or 6 hrs were analyzed by immunoblotting for indicated proteins. Click here for file

Additional file 7 **Regulation of cisplatin-induced apoptosis and necrosis by DNA-PKcs and SSRP1**. A. Regulation of cisplatin-induced apoptosis and necrosis by DNA-PKcs. Free nucleosomes were quantified in culture supernatants (necrosis) and lysates (apoptosis) of DNA-PKcs and control shRNA-expressing A2780 cells treated with various concentrations of cisplatin, from 1.5 to 25 μg/ml for 24 hrs. B. Regulation of cisplatin-induced apoptosis and necrosis by SSRP1. SSRP1 and control shRNA-expressing A2780 cells were treated with various concentrations of cisplatin for 24 hrs. Nucleosomes were quantified as in A. Click here for file
