# Supplementary figures and images for: Concordant and opposite roles of DNA-PK and the "facilitator of chromatin transcription" (FACT) in DNA repair, apoptosis and necrosis after cisplatin
Source: Mol Cancer. 2011 Jun 16;10:74. doi: 10.1186/1476-4598-10-74 (PMC3135565; doi:10.1186/1476-4598-10-74)

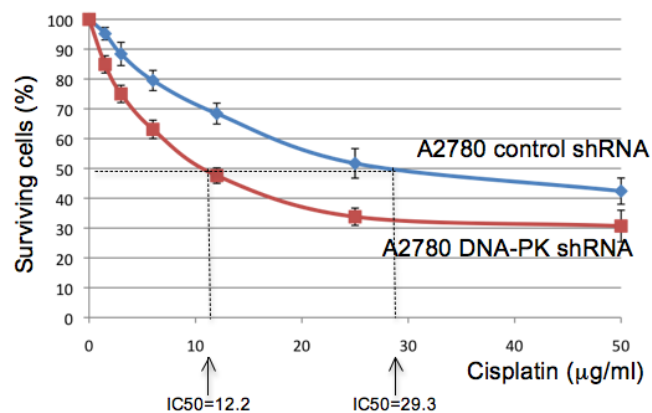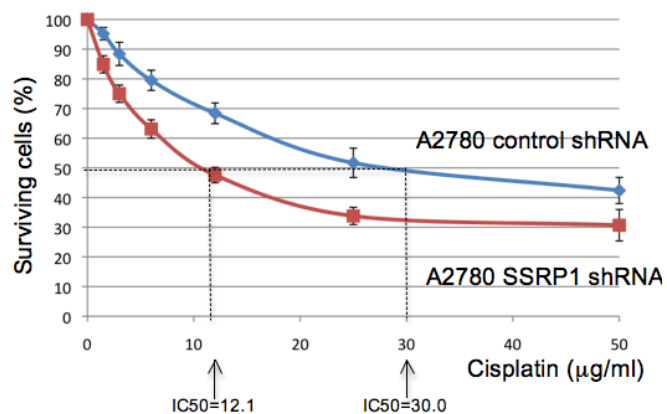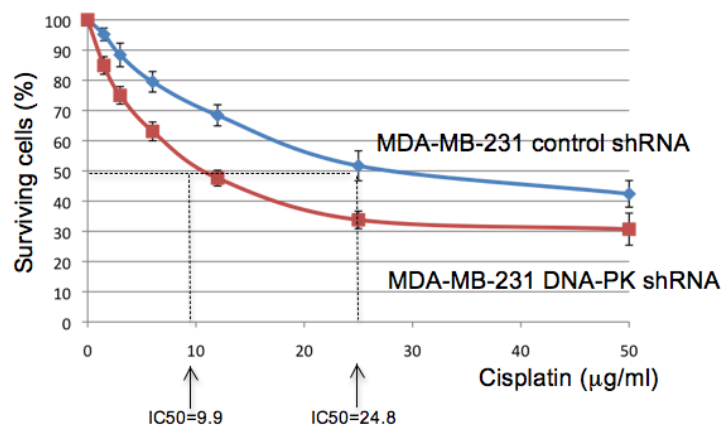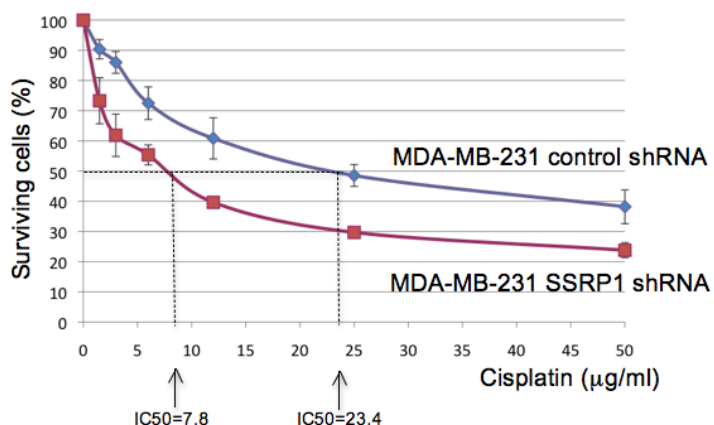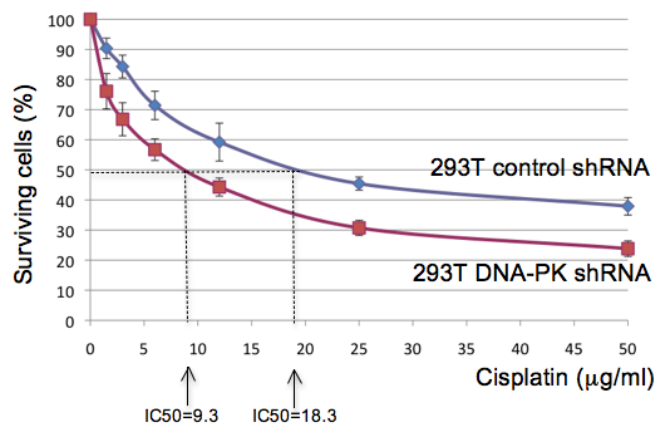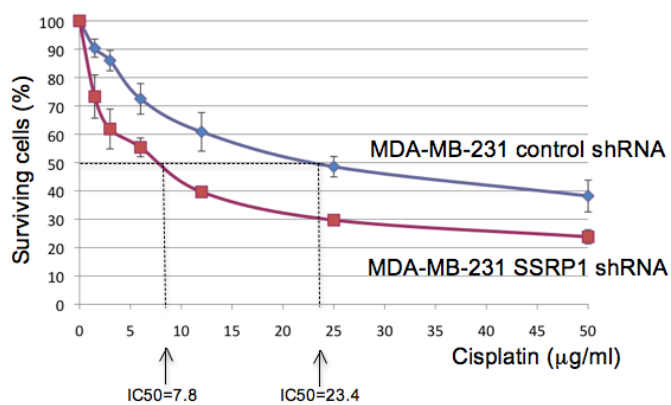

Supplement: Additional file 1 — Silencing the expression of DNA-PKcs or SSRP1 increases cell sensitivity to cisplatin. DNA-PKcs, SSRP1 and control shRNA-expressing A2780, MDA-MB-231 and HEK293T cells were treated with increasing concentrations of cisplatin as indicated. Cytotoxicity was assessed by the MTS assay. [file 1476-4598-10-74-S1.PDF]

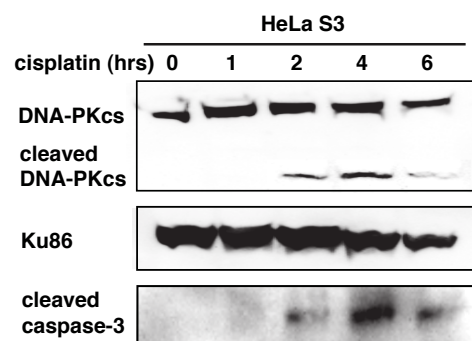

**Additional file 2**  
**Sand-Dejmek et al**

Supplement: Additional file 2 — DNA-PK Regulates cisplatin-induced apoptosis. Extracts from HeLa-S3 cells treated with 100 μg/ml cisplatin for the indicated time were immunoblotted for DNA-PKcs, Ku86 and cleaved caspase-3. [file 1476-4598-10-74-S2.PDF]

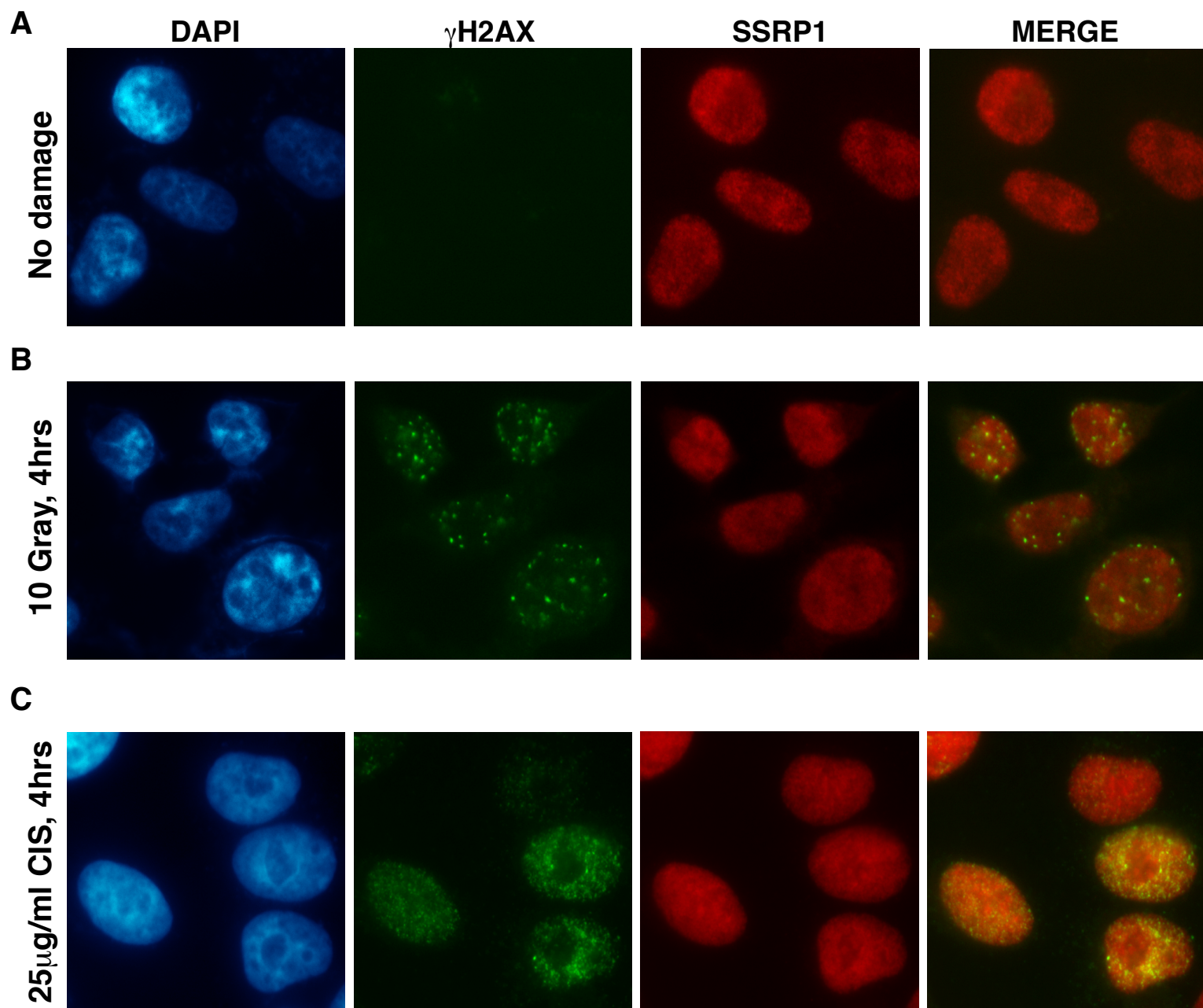

Additional file 3

Supplement: Additional file 3 — SSRP1 does not co-localize with DNA damage-induced γH2AX foci. A2780 cells were immunostained for SSRP1 and γH2AX before or 4 hours after DNA damage. Nuclei were visualized with DAPI. A. Undamaged cells. B. Gamma irradiation (10 Gray). C. Cisplatin treatment (25 μg/ml). [file 1476-4598-10-74-S3.PDF]

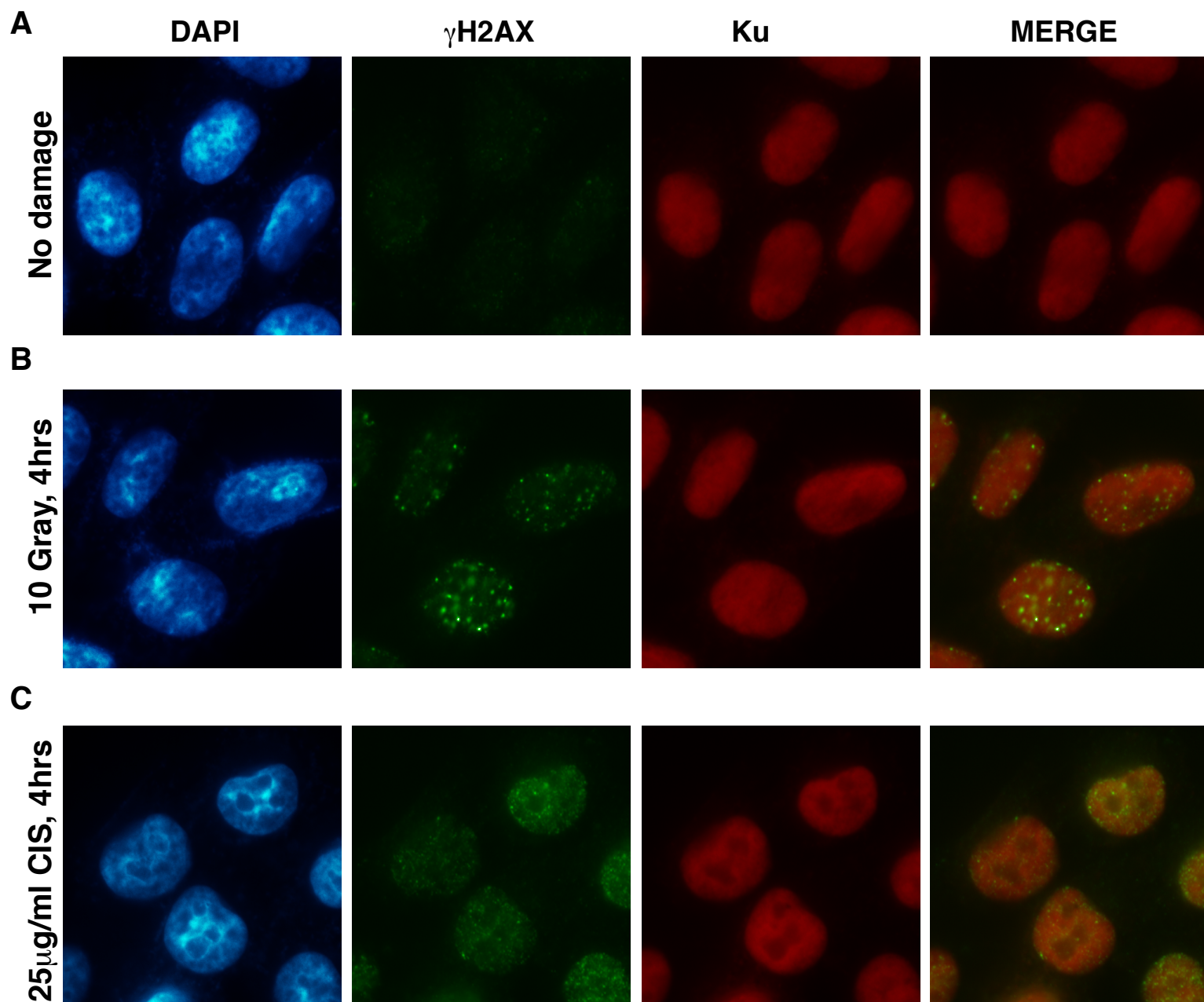

Additional file 4

Supplement: Additional file 4 — Ku86 does not co-localize with DNA damage-induced γH2AX foci. A2780 cells were immunostained for Ku86 and γH2AX before or 4 hours after DNA damage. Nuclei were visualized with DAPI. A. Undamaged cells. B. Gamma irradiation (10 Gray). C. Cisplatin treatment (25 μg/ml). [file 1476-4598-10-74-S4.PDF]

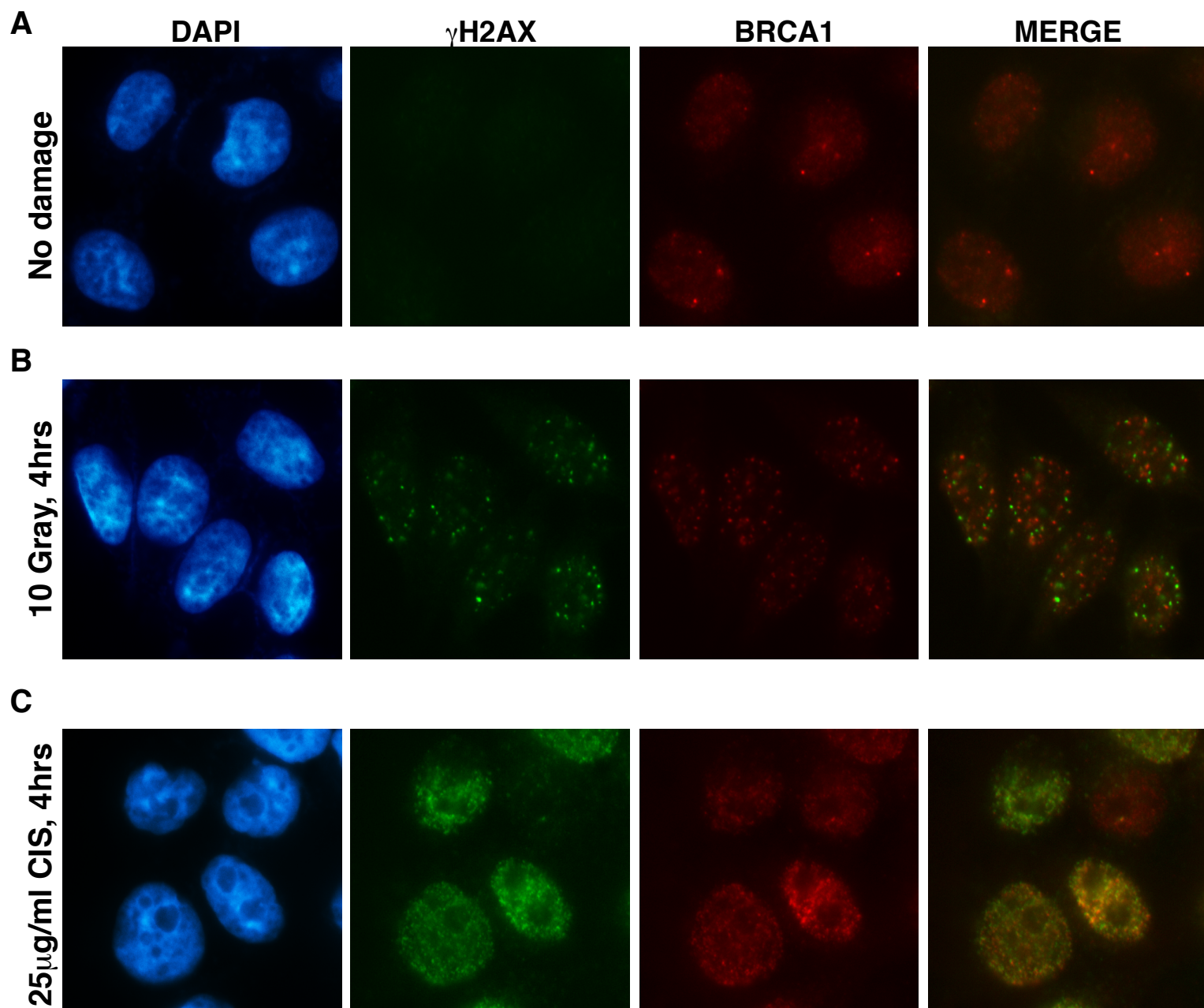

Additional file 5

Supplement: Additional file 5 — BRCA1 co-localizes with DNA damage-induced γH2AX foci. A2780 cells were immunostained for BRCA1 and γH2AX before or 4 hours after DNA damage. Nuclei were visualized with DAPI. A. Undamaged cells. B. Gamma irradiation (10 Gray). C. Cisplatin treatment (25 μg/ml). [file 1476-4598-10-74-S5.PDF]

**a**

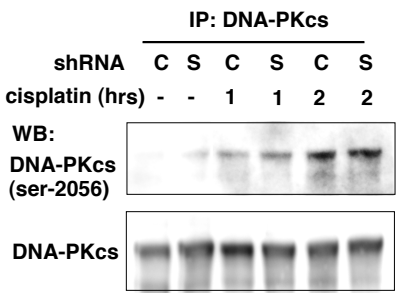

C = Control  
S = SSRP1

**b**

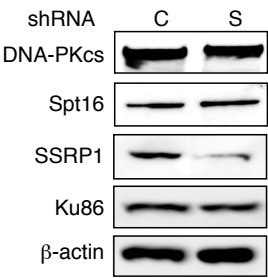

**c**

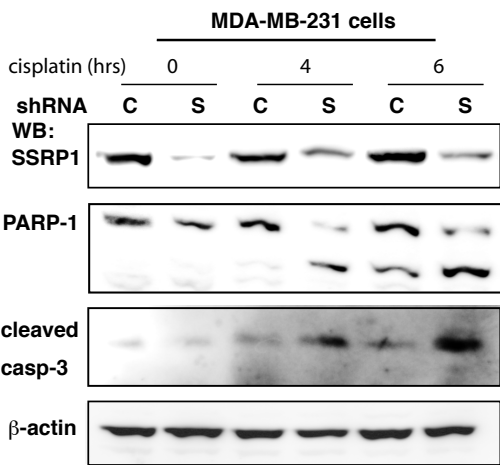

Additional file 6

Supplement: Additional file 6 — Effects of SSRP1 depletion on DNA-PK and apoptosis. A. DNA-PK activation after cisplatin treatment is SSRP1-independent. DNA-PKcs was immunoprecipitated from SSRP1 and control shRNA expressing A2780 cells treated with 100 μg/ml cisplatin for 0, 1 and 2 hours. Immunoprecipitates were analyzed by immunoblotting for DNA-PKcs-Ser2056 and DNA-PKcs. B. SSRP1 knock down does not alter Ku86, Spt16 and DNA-PKcs expression. Whole cell lysates of SSRP1 and control shRNA-expressing A2780 cells were analyzed by immunoblotting for β-actin, Ku86, SSRP1, Spt16 and DNA-PKcs. C. Role of FACT in cisplatin-induced apoptosis in MDA-MB-231 breast cancer cells. Whole cell lysates of SSRP1 or control shRNA-expressing cells treated with 100 μg/ml cisplatin for 0, 4 or 6 hrs were analyzed by immunoblotting for indicated proteins. [file 1476-4598-10-74-S6.PDF]

**A**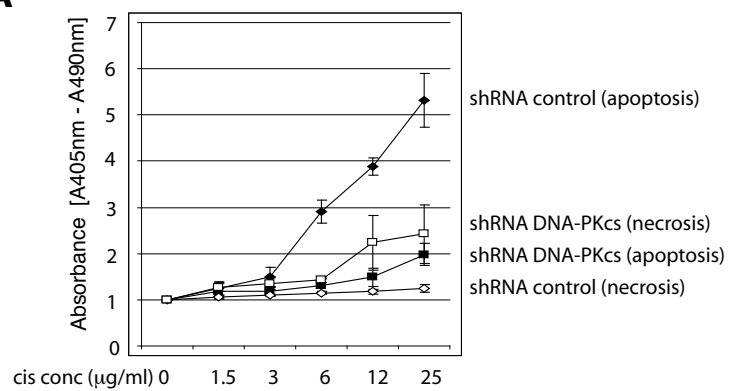**B**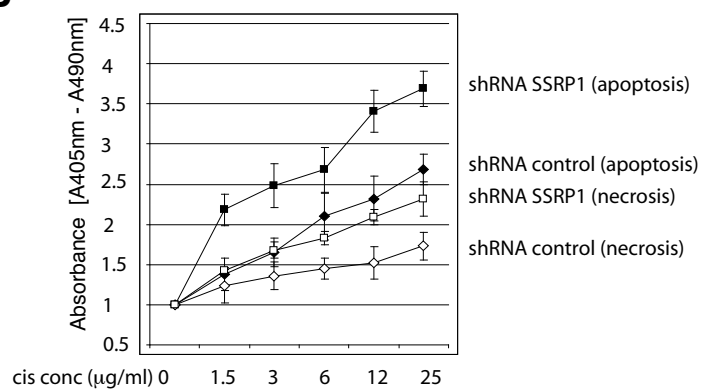**Additional file 7**

Supplement: Additional file 7 — Regulation of cisplatin-induced apoptosis and necrosis by DNA-PKcs and SSRP1. A. Regulation of cisplatin-induced apoptosis and necrosis by DNA-PKcs. Free nucleosomes were quantified in culture supernatants (necrosis) and lysates (apoptosis) of DNA-PKcs and control shRNA-expressing A2780 cells treated with various concentrations of cisplatin, from 1.5 to 25 μg/ml for 24 hrs. B. Regulation of cisplatin-induced apoptosis and necrosis by SSRP1. SSRP1 and control shRNA-expressing A2780 cells were treated with various concentrations of cisplatin for 24 hrs. Nucleosomes were quantified as in A. [file 1476-4598-10-74-S7.PDF]
